# Intraoperative extracorporeal blood purification therapy during major septic vascular surgery

**DOI:** 10.1186/s13054-022-04284-7

**Published:** 2022-12-27

**Authors:** Céline Monard, Philippe Tresson, Antoine Lamblin, Farida Benatir, Xavier-Jean Taverna, Thomas Rimmelé

**Affiliations:** 1 Service d’Anesthésie-Réanimation, Hôpital Edouard Herriot, Hospices Civils de Lyon, Lyon, France; 2 Service de Chirurgie Vasculaire et Endovasculaire, Hôpital Louis Pradel, Hospices Civils de Lyon, Bron, France; 3 EA 7426, PI3 (Pathophysiology of Injury-Induced Immunosuppression), Université Claude Bernard Lyon 1-Biomérieux-Hospices Civils de Lyon, Lyon, France; 4 Centre de Référence des Infections Vasculaire Complexes (CRIVasc Network), Hospices Civils de Lyon, Lyon, France

**Keywords:** Anesthesia, Aortic surgery, Extracorporeal blood purification therapy, Major surgery, Prosthetic infection, Renal replacement therapy, Sepsis, Vascular surgery

The development of complex surgical procedures requires simultaneous development of enhanced intraoperative resuscitation therapies. Procedures such as extracorporeal organ support, usually performed in intensive care units (ICU), could be transferred to operating rooms.

Continuous renal replacement therapy (CRRT) may improve metabolic homeostasis during surgeries with ischemia–reperfusion [1]. Other extracorporeal blood purification therapies (EBP) such as hemoperfusion may decrease surgery-associated inflammation by removing inflammatory mediators from the blood. Cytokine adsorbers have been used safely during cardiopulmonary bypass [2]. Seraph^®^ 100 (Exthera Medical, Martinez, CA, USA) is a new hemoadsorption device designed to adsorb pathogens [3, 4]. Since it decreases the blood pathogen load, it may attenuate sepsis when initiated early after bacterial release in the blood.

Surgery for aortic vascular or endovascular graft infection (VEGI) requires aortic clamping and may cause disseminated pathogenemia, resulting in major inflammation and metastatic infection. This surgery is associated with prolonged postoperative ICU stay (14 ± 12 days), high incidence of complications, and in-hospital mortality may reach up to 50% [5]. We hypothesized that an intraoperative enhanced resuscitation protocol combining CRRT and Seraph^®^ 100 is safe and feasible and could improve outcomes. This letter provides the protocol description and the outcomes of the first patients consecutively treated and discusses the benefits and limitations of this procedure.

After VEGI confirmation, patients were scheduled for surgery with intraoperative CRRT and Seraph^®^ 100. Empirical broad-spectrum antimicrobial therapy was initiated preoperatively, immediately after bacteriological sampling.

In the operating room, general anesthesia was performed according to standard practices. After induction, a dialysis catheter was inserted and continuous venovenous hemodialysis (CVVHD) was initiated with regional citrate anticoagulation (RCA). An EMiC^®^2 hemofilter was used and the Seraph^®^ 100 cartridge incorporated between the blood pump and the hemofilter. Blood flow was set between 100 and 120 mL min^−1^ and dialysate flow between 25 and 30 mL kg^−1^ h^−1^. An ICU nurse, trained in CVVHD and Seraph^®^ 100 use, was in charge of the session monitoring. Dialysate composition, citrate, and calcium replacement were adjusted during surgery as needed. At the end of surgery, blood was returned to the patient before transfer to the ICU where CRRT could be resumed (or not) depending on hemodynamic and metabolic status.

Between December 2021 and May 2022, 6 patients underwent excision of vascular graft and infected tissues associated with in situ reconstruction using cryopreserved arterial allografts. They all received intraoperative CRRT and Seraph^®^ 100. The median [interquartile range, IQR] end-of-surgery lactate and potassium levels were 3.7 [3.2–5] and 4.2 [4.1–4.9] mmol l^−1^. The median [IQR] postoperative ICU length of stay was 6.5 [4.5–10] days. All patients were alive 3 months after the procedure. One patient required a secondary surgical procedure at postoperative day 6 for mesenteric ischemia. Three patients exhibited intraoperative ionized hypocalcemia. No other EBP therapy-related adverse event was observed (Table 1).

**Table 1:** Patient characteristics

Patient	1	2	3	4	5	6
Age (years)	78	18	58	75	61	68
Comorbidities	Diabetes Solid tumor KB infection	Denutrition Multiple ECMO complications (CCA after PE)	Denutrition Ischemic cardiopathy OALL	Hypertension Aorto-enteric fistula	Denutrition Hypertension Solid tumor OALL	Hypertension Diabetes COPD
*Medical history*						
Previous graft	Aortic endograft for mycotic pseudoaneurysm	Ilio-femoral prosthetic graft for bypass rupture	Aorto-bifemoral graft	Aortic endograft for aorto-enteric fistula	Aorto-bifemoral graft	Aorto-bifemoral graft
Preoperative characteristics	Disseminated KB infection	Chronic sepsis, hospitalized in ICU for 4 months	Groin infection with wound leaking	Fistula between endograft and duodenum	Groin infection: drainage and NPWT	Groin and aortic graft infection
*Surgical information*						
Surgery description	Endograft removal with aorto-biiliac CAA reconstruction	Graft removal with in situ CAA reconstruction	Graft removal with CAA reconstruction	Endograft removal with CAA reconstruction + intestinal procedure	Graft removal with CAA reconstruction	Graft removal with CAA reconstruction
Surgery duration (minutes)	259	253	303	396	381	362
Aortic clamping duration (minutes)	80	89	72	71	101	102
*Anesthesia information*						
Red blood cell units (n)	3	2	2	2	2	0
Crystalloids (mL)	8600	3000	6500	7000	11,000	5000
Maximal norepinephrine dose (μg/kg/min)	0.9	0.4	1,7	0.4	0.8	0.4
Worst intraoperative pH	7.14	7.26	7.18	7.29	7.24	7.25
End-of-surgery K^+^ level (mmol/L)	4.3	4.1	5.5	3.3	4.2	5.2
End-of-surgery lactate level (mmol/L)	5.4	3.1	3.8	3.7	5.9	2.9
*Outcomes*						
Postoperative MV duration (hours)	48	5	20	3	48	6
Postoperative RRT duration (h)	30	0	24	16	16	46
Postoperative vasopressors duration (days)	1	0	3	2	3	1
ICU LOS (days)	6	17	4	7	11	3
Hospital LOS (days)	17	146	11	17	20	27
*Adverse events*						
Intraoperative complications	None	None	None	Ionized hypocalcemia 1.06 mmol/L	Ionized hypocalcemia 0.93 mmol/L	Ionized hypocalcemia 1.08 mmol/L
Postoperative complications	At 4 months, relapse of aortic infection with KB	Pursuit of NPWT	None	None	Mesenteric ischemia at POD 6	None
Status at 3 months	Alive	Alive	Alive	Alive	Alive	Alive

Kb, Koch’s bacillus; CAA, Cryopreserved arterial allograft; CCA, Cardiocirculatory arrest; ECMO, Extracorporeal membrane oxygenation; MV, Mechanical ventilation; OALL, Obliterating arteriopathy of the lower limbs; NPWT, Negative-pressure wound therapy; LOS, Length of stay; ICU, Intensive care unit; PE, Pulmonary embolism; POD, Postoperative day; RRT, Renal replacement therapy

The procedure was easily implemented thanks to a productive collaboration between anesthesiologists and intensivists and met broad support from the surgical team as it did not interfere with the surgical procedure and was perceived as improving intraoperative hemodynamic stability. However, it required the presence of a dedicated and trained ICU nurse.

Because vascular clamping is prolonged during VEGI surgeries, patients often present major metabolic disturbances and up to 50% of them require RRT at ICU admission [5]. We initiated intraoperative RRT to target metabolic homeostasis, and none of these patients experienced severe acidosis or hyperkalemia. Importantly, RCA enabled EBP without heparin anticoagulation, which is safer in this surgical context. RRT was discontinued within 48 h after ICU admission for all patients.

The use of Seraph^®^ 100 might have improved the intraoperative hemodynamic stability, as this pathogens’ adsorption device may offer a better control of inflammation. All of intraoperative blood cultures remained negative, and we hypothesized that preoperative administration of antimicrobials decreased their sensitivity. Drug removal through the extracorporeal circuit should be questioned, although no interference with anesthesia was observed.

We report the first cases of intraoperative EBP combining RRT and pathogen hemoadsorption for VEGI surgeries. This treatment is feasible and associated with excellent outcomes compared to what is usually observed. Despite severe preoperative conditions, all patients were alive after 3 months. Intraoperative extracorporeal kidney and immunologic support may be combined in other specific situations including endocarditis, composite tissue allografts, and complex liver surgery. These findings need to be further evaluated in interventional trials.

## Data Availability

The dataset is available from the corresponding author on reasonable request.

## Abbreviations

CRRT: Continuous renal replacement therapy
CVVHD: Continuous venovenous hemodialysis
EBP: Extracorporeal blood purification
ICU: Intensive care unit
IQR: Interquartile range
RCA: Regional citrate anticoagulation
RRT: Renal replacement therapy
VEGI: Vascular or endovascular graft infection
